# A pipeline for local assembly of minisatellite alleles from single-molecule sequencing data

**DOI:** 10.1093/bioinformatics/btw687

**Published:** 2016-12-05

**Authors:** Denye Ogeh, Richard Badge

**Affiliations:** Department of Genetics, University of Leicester, Leicester, UK

## Abstract

**Motivation:**

The advent of Next Generation Sequencing (NGS) has led to the generation of enormous volumes of short read sequence data, cheaply and in reasonable time scales. Nevertheless, the quality of genome assemblies generated using NGS technologies has been greatly affected, compared to those generated using Sanger DNA sequencing. This is largely due to the inability of short read sequence data to scaffold repetitive structures, creating gaps, inversions and rearrangements and resulting in assemblies that are, at best, draft forms. Third generation single-molecule sequencing (SMS) technologies (e.g. Pacific Biosciences Single Molecule Real Time (SMRT) system) address this challenge by generating sequences with increased read lengths, offering the prospect to better recover these complex repetitive structures, concomitantly improving assembly quality.

**Results:**

Here, we evaluate the ability of SMS data (specifically human genome Pacific Biosciences SMRT data) to recover poorly represented repetitive sequences (specifically, GC-rich human minisatellites). To do this we designed a pipeline for the collection, processing and local assembly of single-molecule sequence data to form accurate contiguous local reconstructions. Our results show the recovery of an allele of the non-coding minisatellite MS1 (located on chromosome 1 at 1p33-35) at greater than 97% identity to reference (GRCh38) from the unprocessed sequence data of a haploid complete hydatidiform mole (CHM1) cell line. Furthermore, our assembly revealed an allele of over 500 repeat units; much larger than the reference (GRCh38), but consistent in structure with naturally occurring alleles that are segregating in human populations. This local assembly’s reconstruction was validated with the release of the whole genome assemblies GCA_001297185.1 and GCA_000772585.3, where this allele occurs. Additionally, application of this pipeline to coding minisatellites in the PRDM9 and ZNF93 genes enabled recovery of high identity allele structures for these sequence regions whose length was confirmed by PCR from cell line genomic DNA. The internal repeat structure of the PRDM9 allele recovered was consistent with common human-specific alleles.

**Availability and Implementation:**

Code available at https://github.com/ndliberial/smrt_pipeline

## 1 Introduction

Genome sequencing (especially, of large genomes) has improved greatly in terms of speed and cost as a result of the NGS revolution (Henson *et al.*, 2012). Despite the re-sequencing of the human genome (Ju *et al.*, 2011; Schuster *et al.*, 2010; Wang *et al.*, 2008) and the *de novo* assemblies of the Panda (Li *et al.*, 2010) and Turkey (Dalloul *et al.*, 2010) genomes by purely NGS approaches, the quality of genome assemblies is still greatly affected by the short read length and the errors generated by these technologies (Henson *et al.*, 2012). The presence of high copy number repeats in the genome DNA sequence, severely limits the ability of assembly software (hereafter, assemblers) to infer the relative positions of reads in the genome (Henson *et al.*, 2012). This effect is particularly acute for very short reads (<100 bp) and highly repetitive genomes (Henson *et al.*, 2012). Thus, there is a need for assemblers that implement novel strategies for dealing with such difficulties in NGS generated data. In addition to repeats, the possibility of systematically incorrect base calling errors can lead to reads being more similar to the wrong location in the genome, reducing assembly contiguity (Salzberg *et al.*, 2012; Schatz *et al.*, 2010). Although NGS technologies now generate large datasets of short sequence reads with higher coverage to compensate for the reduced connectivity between reads and to improve assembly, repetitive sequences that are longer than NGS read lengths still cannot be resolved solely by higher coverage, resulting in gaps in assemblies being biased towards repetitive regions (Salzberg *et al.*, 2012; Schatz *et al.*, 2010). The challenge of assembling genomic sequences from NGS data at particular regions means that SMS technologies (particularly, Pacific Biosciences SMRT) with much longer read lengths become highly attractive. Recent studies have reported the use of long reads in improving and validating assemblies (English *et al.*, 2012; Huddleston *et al.*, 2014; McCoy *et al.*, 2014) and at high coverage (>90-fold), high quality assemblies of moderate size genomes have been generated from single-molecule long reads using pre-assembly error correction methods (English *et al.*, 2012; Huddleston *et al.*, 2014; McCoy *et al.*, 2014). Despite the high indel error rate associated with PacBio SMRT technology, these data have been shown to be effective in traversing common repeats during assembly (Huddleston *et al.*, 2014).

## 2 Methods

### 2.1. Dataset

Publicly available single molecule long-read sequencing data from human DNA (CHM1 htert cell line) was downloaded from PacBio's blog (http://datasets.pacb.com/2014/Human54x/fast) via a custom Perl script (https://github.com/ndliberial/smrt_pipeline). The CHM1 haploid cell line was derived from a hydatidiform mole, a kind of abnormal pregnancy in which an egg without nuclear DNA is fertilized by an ordinary sperm (http://www.bio-itworld.com/2014/6/30/hunt-new-human-reference-genome.html). Because the sperm doubles its DNA, two identical copies of each chromosome is generated in every cell. Hydatidiform moles are rare, yet CHM1 is an established and widely used haploid cell line (Fan *et al.*, 2002).

### 2.2. Analysis scripts

Custom Perl wrapper scripts (see github repositories for listing) were utilized in each stage of the pipeline, to provision, run and manage the output of third party software.

### 2.3. Third-party softwares

Third-party tools include; Celera assembler 8.2 (Myers *et al.*, 2000), Burrows-Wheeler Aligner (BWA 0.79) (Li, 2013), LAST v4.75 (Kiełbasa *et al.*, 2011), Tandem Repeat Finder (TRF 4.09) (Benson, 1999), UCSC Genome Browser (Kent *et al.*, 2002), BLAST-like alignment tool (BLAT) (Kent, 2002), RepeatMasker (Smit *et al*., 2013), Basic Local Alignment Search Tool (BLAST) (Altschul *et al.*, 1990), Perl v5.18.2, BioPerl 1.6.924 (http://search.cpan.org/dist/BioPerl/), Samtools 0.1.19 (Li *et al.*, 2009), *In-Silico* PCR (https://genome.ucsc.edu/cgi-bin/hgPcr) and IGV v2.3 (Robinson *et al.*, 2011).

### 2.4. Hardware resources

Linux (Ubuntu 14.04 LTS) workstation or a High Performance Computing (HPC) cluster with Internet connection and a minimum display resolution of 800 × 600 dpi.

## 3 Results

### 3.1 Description of datasets

The dataset used in this study was generated from single molecule long-read sequencing of the well-studied human cell line (CHM1 htert) to ∼54× coverage (http://www.pacb.com/blog/data-release-54x-long-read-coverage-for/).

### 3.2 Analysis and assembly pipeline

**Fig. 1:**
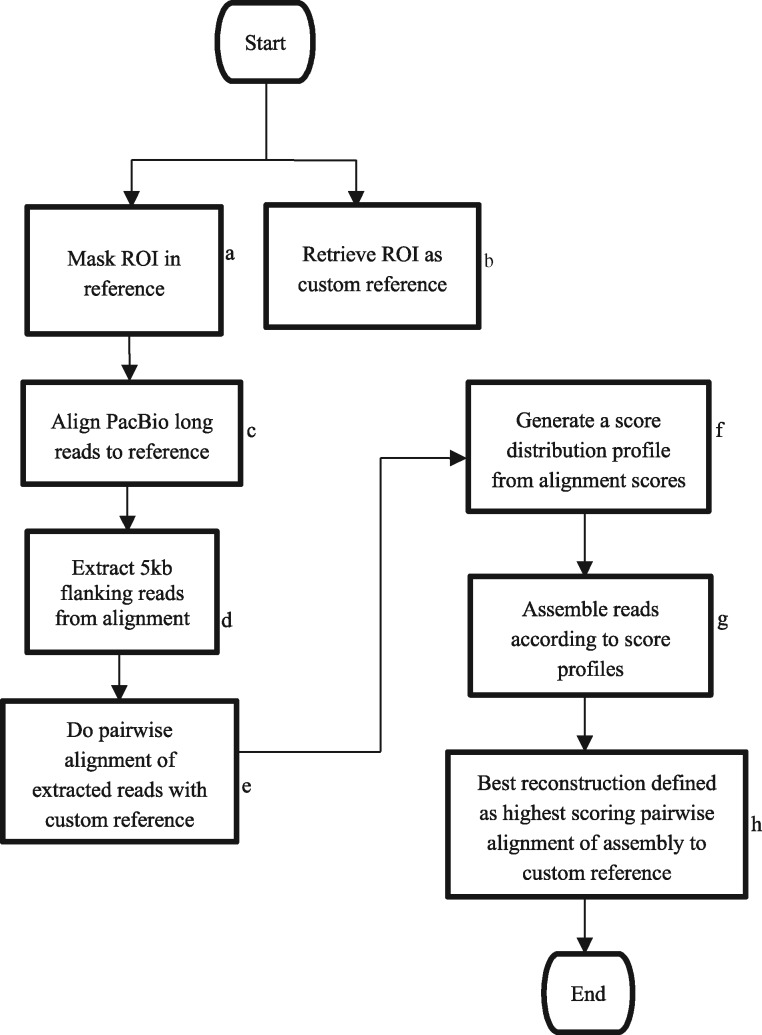
Analysis and assembly pipeline workflow

In order to efficiently retrieve, process, assemble and analyze the dataset for presence/absence of the repetitive DNA of interest, we designed and implemented the pipeline illustrated (Fig. 1). The pipeline incorporates Perl scripts for data download and downstream analysis. Also included in the pipeline are third-party assembly programs, managed by Perl wrapper scripts. Arrows indicate progression from the start to the end of the analysis and assembly process. Each stage indicated by a blue rectangle must be completed in order for the next stage to commence.

### 3.3 Mapping repetitive DNA

Using primers 5′-GCTTTTCTGTGATGAGCCTTGATG-3′ and 5′-AGAAGCATATGCAACCCATGAGG-3′ for MS1 (Gray and Jeffreys, 1991) and 5′-TGAGGTTACCTAGTCTGGCA-3′ and 5′-ATAAGGGGTCAGCAGACTTC-3′ (Berg *et al.*, 2010) for PRDM9, Regions of Interest (ROI) were extracted from the genome reference assembly (GRCh38) using *In-Silico* PCR to generate custom reference ‘bait sequences’ (Fig. 1b). The ROIs were repeatmasked (Quinlan and Hall, 2010) (Fig. 1a) to generate a masked reference. The mapping of long PacBio^®^ reads to these masked reference bait sequences (Fig. 1c) and the subsequent extraction of 5 kb flanking reads across the ROI (Fig. 1d) generated spanning reads which potentially held information to enable the reconstruction of the repeat array. Using LAST (Kiełbasa *et al.*, 2011), a score distribution profile of reads with sufficient identity to the region was generated (Fig. 1e, f). The reads in each score distribution bin were assembled (Fig. 1g) using Celera 8.2 (Myers *et al.*, 2000). The highest scoring pairwise alignment of assembly to the reference was used to recover the tandem repeat array (Fig.1h).

**Fig. 2:**
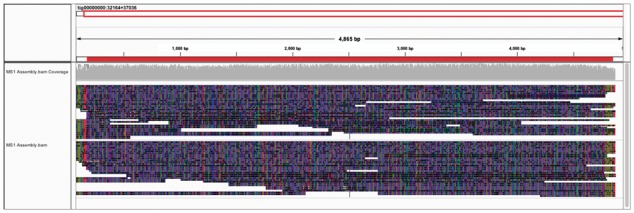
IGV visualization showing mapping of assembly contributing reads to the assembly consensus. The minisatellite array (red) extends through the majority of the assembly

To illustrate that our local assembly pipeline integrates multiple array spanning reads as well as reads that terminate within the MS1 array we visualized their mapping using IGV (Robinson *et al.*, 2011). 82 individual reads contribute to the assembly shown in Figure 2. The red coloured region indicates the location of the minisatellite repeat array.

### 3.4 Copy number variation between local assembly, GRCh38 and PacBio-based global genome assembly

We used Tandem Repeat Finder (Benson, 1999) to determine the number of repeat units in the various assemblies. As shown in Table 1 below, our assembly for MS1 and PRDM9 showed >95% identity to both the reference and PacBio assemblies, where aligned. Repeat copy number is 10× of the reference with an array size of over 5 kb. The validity of our analysis and assembly pipeline was proven with the agreement in copy number and high sequence identity between our local assembly which is derived from reads generated using an earlier sequencing chemistry (P5-C3) as compared to PacBio's global genome assembly derived from reads generated using the more recent P6-C4 sequencing chemistry (http://www.ncbi.nlm.nih.gov/Traces/sra/?study=SRP044331).

**Table 1.** Summary statistics of minisatellite assembly

Repeat	Copy number (Ref.)	Copy number (Ass.)	Copy number (PacBio.)	Identity of assembly to reference (%)	Identity of assembly to PacBio (%)	*In-Silico* PCR size (Ref.)	*In-Silico* PCR size (Ass.)	*In-Silico* PCR Size (PacBio)
MS1	53.8	523.8	513.8	97.1	96	1.2	5.5	4.5
PRDM9	12.5	12.5	12.5	96.8	98.9	1.9	1.9	1.9

### 3.5 Minisatellite array structures?

**Fig. 3:**
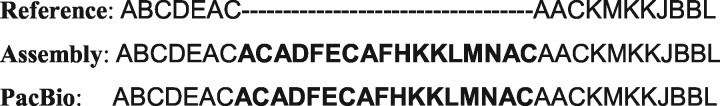
Comparison of the internal structures of a known MS1 allele, the local assembly generated by our pipeline and PacBio's global genome assembly. Bold letters indicate the missing sequence in the reference, which is recovered by our local assembly as well as PacBio’s global genome assembly

**Fig. 4:**
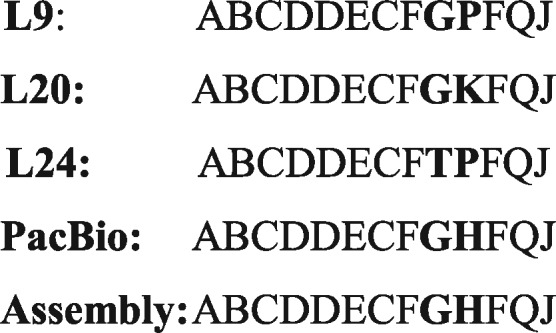
Comparison of the internal structures of known (L9, 20 and 24) (Berg *et al.*, 2010) PRDM9 alleles, the local assembly generated by our pipeline and PacBio's global genome PRDM9. The bold letters indicate variation in allele structure

Previous work done by Gray and Jeffreys (Gray and Jeffreys, 1991) in identifying variations in the MS1 9bp repeat sequence identified 19 variations occurring as a result of base changes from the consensus repeat unit sequence. These 19 variant types were coded as A to S, following the scheme developed in Minisatellite Variant Repeat PCR (MVR-PCR) mapping (Gray and Jeffreys, 1991). Figures 3 and 4 show the allelic structures at MS1 and PRDM9 for the GRCh38 reference, PacBio assembly and our assembly.

In general, reconstruction of minisatellite alleles using our local assembly pipeline recovers biologically feasible alleles (Figs. 3 and 4), which are validated by the PacBio genome assembly.

### 3.6 The impact of alignment programs on sequence alignment

The type of alignment program was noted as affecting the quality of the alignment and particularly the number of reads included in the assembly. This was observed in the final output from the pipeline where, for example, assemblies derived from BLASR (Altschul *et al.*, 1990) mapping of the coding minisatellite in the (ZNF93 gene) failed to recover the ROI whereas, assemblies derived using BWA-MEM (Li, 2013) on the same region, accurately recovered the ZNF93 Zinc Finger array (data not shown)

## 4 Discussion

In an effort to reconstruct repeat sequences that have been, hitherto, difficult to sequence and assemble with Sanger and short read NGS technologies, we developed software embedded within an analysis and assembly pipeline for the acquisition, filtering, and assembly of single molecule long-read sequencing reads (particularly, PacBio). Application of this pipeline to example minisatellites from coding (PRDM9) and non-coding (MS1) DNA, showed that the approach was effective in recovering minisatellites alleles with over 95% identity to reference where aligned and perfect recovery of internal repeat variant interspersion patterns (Figs. 3 and 4).

Analysis of CHM1 htert—a haploid cell line that is otherwise karyotypically normal, offered the opportunity to simplify assembly by only drawing reads from a single allele. It remains to be seen whether this approach is suitable for reconstruction of repeat structures in diploid regions. We suggest that with sufficient read depth and consensus accuracy, phasing of haplotypes to partition reads between chromosome of origin is feasible. Subsequently our local assembly pipeline can be applied to the partitioned reads, as shown for haploid data.

The choice of a score distribution bin approach allowed retention of long, lower identity alignments which provide contiguity and which at sufficient coverage are expected to yield an accurate consensus. This expectation results from the knowledge that long reads and the majority random indel error mode of the PacBio system means that high identity short alignments can contain less information about repeat structures, than longer lower identity alignments. Also, it is hypothesized that short high identity reads that introduce assembly noise will be efficiently removed by this strategy, while maximizing the recovery of informative reads. Our results suggest that these ideas are plausible. LAST (Kiełbasa *et al.*, 2011) was chosen for performing alignments because the running time scales in a linear (rather than quadratic) fashion based on the sequence length, important when aligning long reads and long contigs. LAST (Kiełbasa *et al.*, 2011) can also be further tuned to optimize for long, weak alignments.

Using the MVR-PCR coding scheme and a custom Perl script to implement edit distance assignment of sequences to repeat types, we showed a 5′ and 3′ consistency in allelic structure between all three (3) assemblies. Whilst the reference (GRCh38) contains a gap, our local assembly and PacBio′s genome assembly both show a recovery of missing and novel repeat units (Fig. 3). Also, the structure of the recovered PRDM9 allele showed consistency with known human-specific alleles (Fig. 4).

Given the accurate representation of coding minisatellites in assembly, and the consistency seen at both the 5′ and 3′ ends of non-coding minisatellites with known allele structures, as described by MVR-PCR mapping, this analysis suggests that our algorithm could be used for the characterization of repetitive sequences that are collapsed or entirely missing in human genome reference sequences. Another benefit of our approach is that local assembly of regions of interest is computationally much less resource intensive than whole genome assembly, and thus accessible to more researchers.

Further validation of assembly structure could be achieved using alternative single molecule sequencing technology such as Oxford Nanopore’s MinION system.

## 5 Conclusion

In conclusion, our study has shown that with single molecule sequencing and long read technology, repetitive sequences (specifically, minisatellites), usually poorly represented in published genome assemblies can be characterized using customized software pipelines, scalable for the analysis of single molecule long reads. Furthermore, the potential for this analysis pipeline to be used for gap closure in reference sequences using high coverage long read data is evident.
